# Smartphone-Based Accurate Analysis of Retinal Vasculature towards Point-of-Care Diagnostics

**DOI:** 10.1038/srep34603

**Published:** 2016-10-04

**Authors:** Xiayu Xu, Wenxiang Ding, Xuemin Wang, Ruofan Cao, Maiye Zhang, Peilin Lv, Feng Xu

**Affiliations:** 1 The Key Laboratory of Biomedical Information Engineering of the Ministry of Education, School of Life Science and Technology, Xi’an Jiaotong University, Xi’an 710049, P. R. China; 2 Bioinspired Engineering and Biomechanics Center (BEBC), Xi’an Jiaotong University, Xi’an 710049, P. R. China; 3 Department of Endocrinology and Metabolism, Fourth Military Medical University, 169 West Changle Road, Xi’an, Shaanxi 710032, P. R. China

## Abstract

Retinal vasculature analysis is important for the early diagnostics of various eye and systemic diseases, making it a potentially useful biomarker, especially for resource-limited regions and countries. Here we developed a smartphone-based retinal image analysis system for point-of-care diagnostics that is able to load a fundus image, segment retinal vessels, analyze individual vessel width, and store or uplink results. The proposed system was not only evaluated on widely used public databases and compared with the state-of-the-art methods, but also validated on clinical images directly acquired with a smartphone. An Android app is also developed to facilitate on-site application of the proposed methods. Both visual assessment and quantitative assessment showed that the proposed methods achieved comparable results to the state-of-the-art methods that require high-standard workstations. The proposed system holds great potential for the early diagnostics of various diseases, such as diabetic retinopathy, for resource-limited regions and countries.

Retinal vasculature changes have been associated with various eye diseases (*e.g.*, diabetic retinopathy) and systemic diseases (*e.g.*, hypertension), which manifest themselves on the retina by altering vessel topological features, such as vessel width and tortuosity^1^,^2^,^3^,^4^,^5^,^6^. Analysis of retinal vessel thus holds great potential to assist the early diagnostics and treatment of these diseases. This is of particular importance for resource-limited regions and countries because retinal vessel is the only part of human blood circulation that can be optically and non-invasively observed *in vivo*, making it relatively easy, safe, and cost-effective^7^,^8^. Diabetic retinopathy (DR), for instance, is a severe complication of diabetes mellitus that can be diagnosed and treated in its early stage to prevent from blindness via regular retinal screening with fundus image. It is estimated that 75% of people with DR live in developing countries (*e.g.*, China and India), where most of the patients are not even diagnosed due to the lack of medical resources and well-trained clinicians^9^. It is also pointed out that the screening program for DR at developing countries should be cost-effective and the decision-making should be automatic in order to reduce workload^9^. Thus there is an urgent need for low-cost, easy-access systems, *i.e.*, point-of-care (POC) diagnostic systems, which are able to provide automatic or semi-automatic diagnostics of diseases, such as DR, in resource-limited settings.

Although significant efforts have been put in the establishment of an automatic POC diagnostic system for resource-limited settings, there are still several important challenges. First of all, the conventional fundus camera used to capture a retinal image, called desktop fundus camera (DF-camera), is bulky and costly, making it not portable and beyond affordability for resource-limited settings. Recently, the rapid development of technologies has driven the advent of various low-cost handheld fundus cameras (HF-camera), providing an alternative option for retinal image acquisition in resource-limited settings. Some of the popular prototypes, including some commercialized devices, attach external optics to a smartphone to capture and store fundus image**s**, greatly reducing the cost and increasing the portability of the device^10^,^11^,^12^. Yet the drawbacks of HF-camera include smaller field-of-view (FOV) and lower image quality as compared to DF-camera. Second of all, the lack of well-trained clinicians and specialists in resource-limited settings indicates an urgent need for on-site automatic diagnostics and decision-making, which is also desired in healthcare cost. containment. Towards this angle, enormous automatic or semi-automatic diagnostic systems based on retinal image analysis have been developed^13^,^14^,^15^,^16^,^17^,^18^,^19^,^20^,^21^,^22^,^23^,^24^,^25^. However, methods with high accuracy often target at high quality images captured with DF-cameras. Moreover, these methods often utilize complicated algorithm designs, such as large-scale data training on high quality images, and are implemented in high-standard workstations. Although there are a few attempts in the establishment of a portable POC diagnostic system^12^,^26^, they were only tested on public databases consisting of high-quality fundus images captured with DF-cameras. Therefore, there is still an unmet need for automatic systems working with low quality fundus images as obtained by the emerging HF-camera. The desired algorithms for a POC diagnostic system should be not only accurate and robust enough to work at resource-limited settings, but also fast and simple enough to be implemented in a portable device (*e.g.*, smartphone).

In this study, we developed a fully automatic retinal image analysis system that can deal with low quality images captured with a HF-camera combined with a smartphone (iExaminer, Welch-Allyn Inc., Skaneateles Fall, NY, USA). This system can read a fundus image, segment retinal vessels, analyze individual vessel width, and store or uplink the results. More specifically, we developed a visual saliency based vessel segmentation method and also a graph-theoretic vessel width measurement method. These two methods were compared with existing methods on high quality fundus images, and also tested on low quality clinical images taken with a smartphone. At last, the proposed system was implemented independently in a smartphone app, which provides a user-friendly interface for image acquisition, test analysis, and result management.

## Results and Discussion

**Figure 1:**
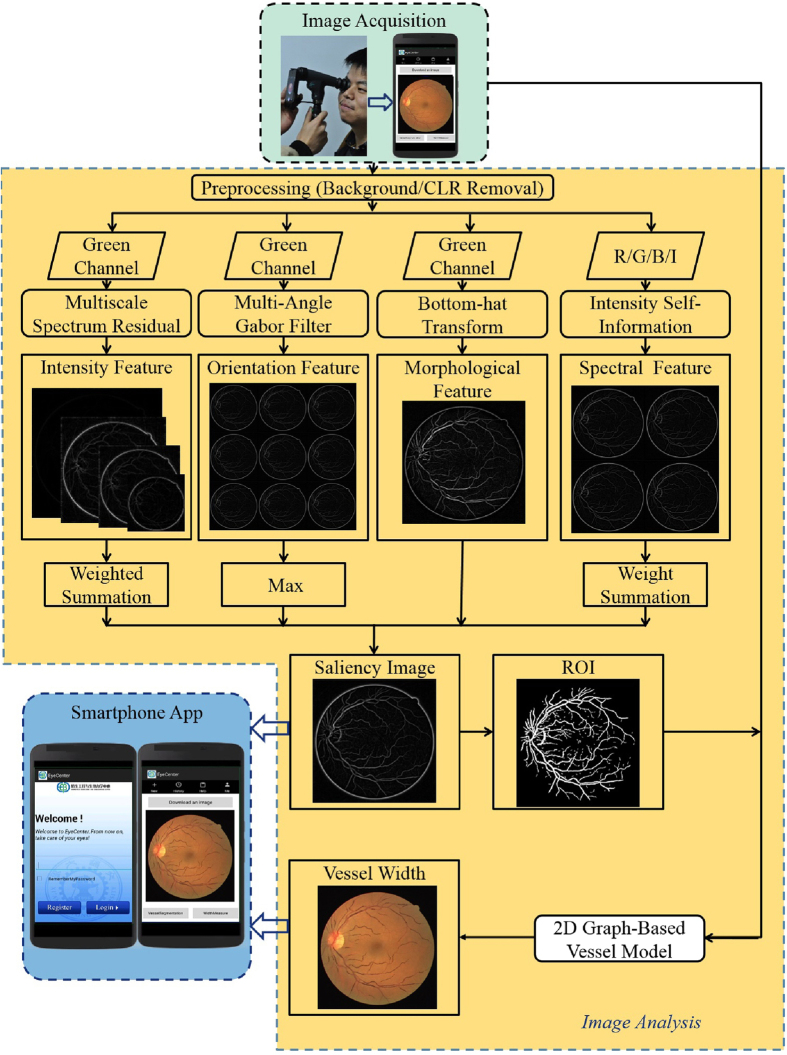
Flowchart of the proposed POC diagnostic system for resource-limited settings. A fundus image is acquired and stored in a smartphone app at resource-limited settings. Then blood vessels are detected and measured inside the app. At last, the results are displayed and saved.

**Figure 2:**
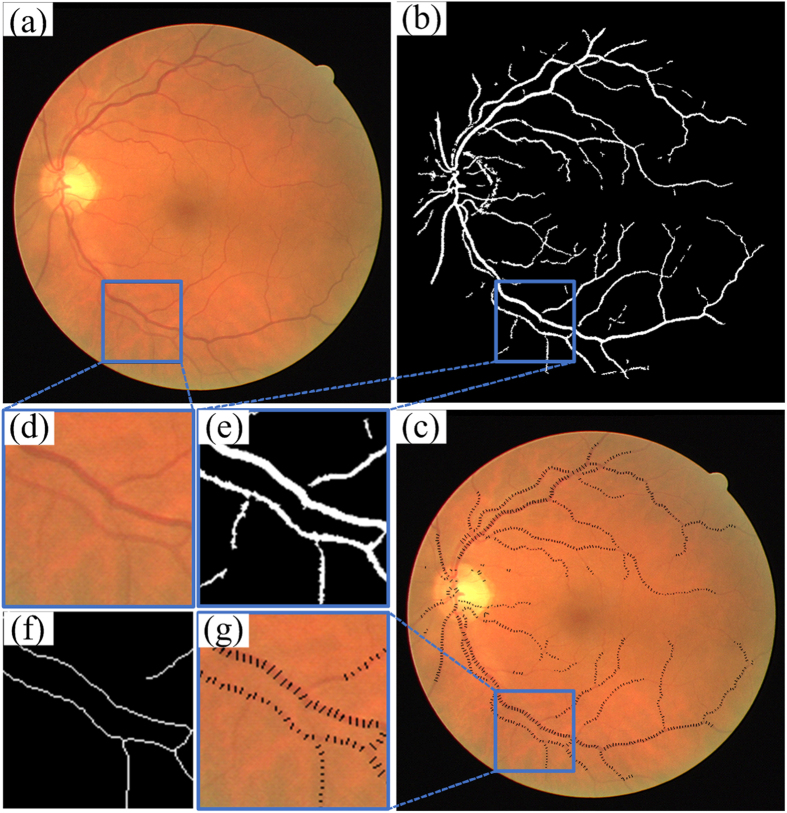
Illustration of the image analysis results. (**a–c**) Original fundus image, vessel segmentation result, and vessel width measurement. (**d–g**) Inset view of original fundus, vessel segmentation, vessel skeleton, and vessel width measurement.

An overview of the whole system is given in Fig. 1. The visualized results of proposed method is given in Fig. 2. Taking a typical image in the DRIVE (Digital Retinal Image for Vessel Extraction) dataset as an example, the results are demonstrated in detail. Starting from the original fundus image (Fig. 2a), the blood vessels are detected (Fig. 2b). We can see the vessel segmentation method was able to detect most of the large vessels, but it missed some of the fine vessels. It also showed high false positives around the optic disc. Then the vessel width was measured at locations where the blood vessel was detected (Fig. 2c). We can see that both large and fine vessels were accurately measured. Figure 2d–g show the inset view of the original fundus image, vessel segmentation, vessel centerline, and vessel width measurement. Specifically, Fig. 2f shows the vessel centerline used in the two-dimensional graph construction, in which the vessel segments with a length smaller than certain pixels were excluded from further vessel width measurement.

**Figure 3:**
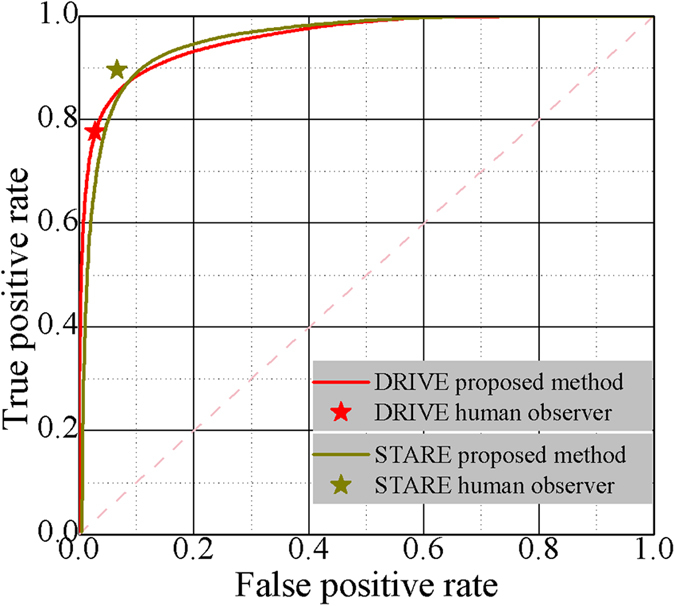
ROC curve for vessel segmentation on the DRIVE and STARE databases. The AUC is 0.9585 for DRIVE and 0.9590 for STARE.

To quantitatively assess the performance of the vessel segmentation algorithm, we evaluated the proposed method on the DRIVE and STARE (Structured Analysis of the Retina) databases by comparing the proposed method with existing methods in Table 1 (Hoover *et al*.^15^, Jiang *et al*.^18^, Staal *et al*.^19^, Mendonca *et al*.^20^, *etc.*). The average accuracy (Acc), sensitivity (SN), and specificity (SP) obtained from our method are 0.933, 0.786, and 0.955 for the DRIVE database, and 0.920, 0.825, and 0.931 for the STARE database, respectively. Even though the proposed method was implemented in a Java Android platform for a smartphone, it showed comparable results with the state-of-the-art methods implemented in standard computer workstations. The running time using the proposed method is ~118 seconds for an image from the DRIVE database (565 × 584 RGB color image) and ~130 seconds for an image from the STARE database (700 × 605 RGB color image). To show the overall performance of the method as the threshold varies, we assessed the receiver operating characteristic (ROC) curve of the proposed method on DRIVE and STARE databases (Fig. 3). For the DRIVE database, the proposed method showed results comparable with human observer (with the red star on the red line), while for the STARE database, the human observer showed a slightly better result than that from the proposed method (with the green star to the left up corner of the green line). The area under the ROC curve (AUC) for DRIVE and STARE were 0.9585 and 0.9590, respectively.

To assess our method for measuring the vessel width, we evaluated the proposed method on the REVIEW database and compared the performance with various existing methods (Table 2 and Table 3). The proposed two-dimensional graph method showed comparable results with the state-of-the-art methods in mean and standard deviation of average vessel width and mean and standard deviation of individual differences. Specifically, compared with the three-dimensional method, the proposed method showed a great improvement in running time. For an image of size 2160 × 1440, the three-dimensional method took 41 seconds to solve all graphs on a standard computer workstation (3.40GHz Intel^®^ Core™ i7-3770 CPU with 8 GB of RAM) and the two-dimensional graph took around 90 seconds to solve all graphs on a smartphone (Qualcomm snapdragon 801, 2.5GHz, RAM 2 GB).

**Figure 4:**
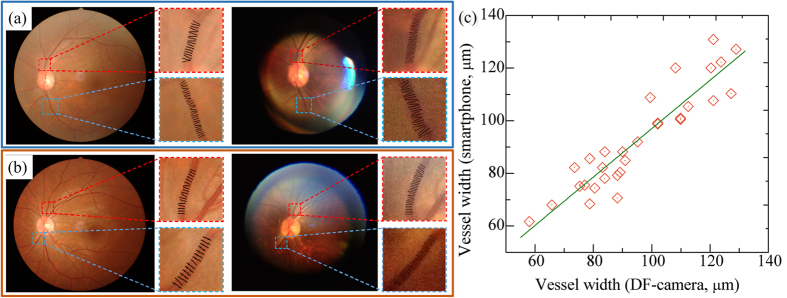
Test on clinical images. (**a**) Visualization of vessel width measurement on high quality image (left) and low quality image (right) of subject one. (**b**) Visualization of vessel width measurement on high quality image (left) and low quality image (right) of subject two. (**c**) The scatter plot of vessel widths by the smartphone with respect to vessel widths by the DF-camera. The Pearson’s correlation is 0.922.

**Figure 5:**
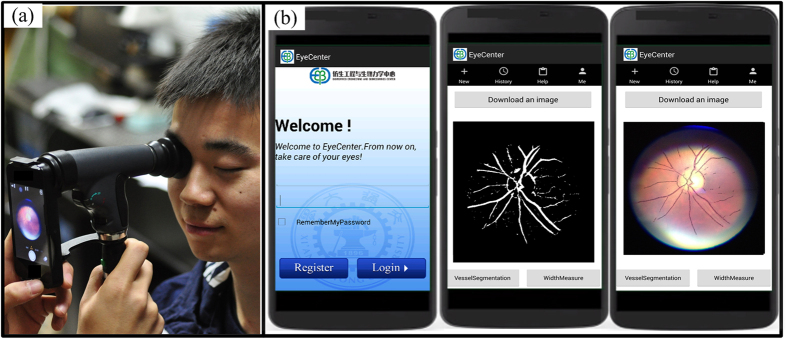
Illustration of the POC diagnostic system and its app. (**a**) Image acquisition. (**b**) Screenshots of the retinal vessel analysis app.

To assess the ability of the proposed system to work as a POC diagnostic tool, we evaluated the performance of the proposed method on low quality clinical images acquired with a smartphone (Fig. 4). For all images, the blood vessels were segmented with the proposed method. To assess the consistency of width measurement, individual vessel segment from the high quality image and its counterpart from the low quality image were selected and measured. For each image, the three widest vessel segments around the optic disc were manually selected by one observer and visually checked by a second observer to guarantee the validity of the vessel, resulting in 30 pairs of vessel segments in total. For each selected vessel, the average vessel width was calculated and adjusted to physical unit (μm) for comparison. The visualized results are given in Fig. 4a,b, in which the left images are high quality image**s** and the right images are the low quality counterparts from the same participant. As can be observed, the low quality images showed smaller FOV, lower image resolution, and poorer contrast compared with their high quality counterpart. To quantitatively assess the algorithmic consistency between high quality images and low quality images, we performed the paired student’s *t*-test and found no significant difference between the two measurements (with a *p*-value of 0.07 in the paired *t*-test). The scatter plot shows good agreement between the two measurements with a Pearson’s correlation of 0.922 (Fig. 4c). These results indicate that the proposed method can give consistent results on high quality image and low quality image. Therefore, the developed system holds great potential as a POC diagnostic tool at resource-limited settings. A user-friendly app was developed to facilitate on-site image acquisition, analysis, and management (Fig. 5). Figure 5a shows the image acquisition and Fig. 5b shows screenshots of the app, which are the home screen, a low quality image after vessel segmentation, and a low quality image after width measurement, respectively.

Retinal vasculature analysis is important for the early diagnostics of various microvascular diseases, as the changes in retinal vessel usually precede the advent of other signs, making it a potentially useful biomarker. However, the manual segmentation and measurement is extremely tedious, difficult, and prone-to-error. The proposed system is proved to be a valuable tool for retinal vasculature analysis at resource-limited settings. Validation on various public databases indicated that this system showed comparable results to the state-of-the-art methods that are complex in design and require high standard workstations as platforms. Testing on low quality images captured by smartphone further confirmed that the proposed system was able to handle low quality images taken at resource-limited settings.

POC diagnostics at resource-limited settings puts high requirements on the on-site diagnostic tools. In smartphone-based image analysis, the first challenge is the ability to deal with low quality images taken with HF-camera. In this study, we not only showed that the proposed system was able to deal with low quality images, but also showed that the performance on low quality images was consistent with the performance on their counterpart high quality images. This is important not only because high quality images are regarded as golden standard and almost all clinical studies are based on high quality images, but also because it provides the patients with a wider choice in later clinical visits. Another important challenge in POC diagnostics is the large variety in test samples. Human retina and retina images show large diversity in background color and vascular geometry because of systemic, environmental and genetic factors^27^. In this respect, one advantage of the proposed system is its independence from any training data, making it readily applicable to unknown images obtained at resource-limited settings. A third challenge in POC diagnostics is its requirements on real-time analysis. Taking advantage of the smartphone, we were able to achieve real-time analysis by keeping the whole test inside a smartphone, including image loading, analysis, and result display. Even though it takes ~3 minutes to finish a test, it is still able to provide on-site and real-time analysis, which is important for resource-limited settings where well-trained clinicians and tele-medicine are not always available.

Although the proposed system has proved to be promising for the detection of within-subject follow-up changes of retinal vasculature, which is important for individual monitoring of disease progression, the between-subject differences also need to be addressed. As discussed above, human retina and retina images show large diversity in background color as well as vascular characteristics, meaning the baseline for health and disease may vary greatly between individuals with different ethnicity, age, etc. Future studies will focus on how these between-subject variabilities can be appreciated.

## Conclusion

In this study, we developed a smartphone-based retinal image analysis system targeting at POC diagnostics at resource-limited settings. This system shows comparable performance with the state-of-the-art methods with a much lower computational complexity. This system can be further combined with commercialized HF-cameras for POC diagnostics or large population screening at resource-limited settings. Future work will include studies on between-subject vascular variability and implementing this algorithm in a POC diagnostic system for the early diagnostics of DR.

## Materials and Methods

### Public database

The vessel segmentation method was evaluated on two popular public databases, which are widely used for the evaluation of retinal vessel segmentation methods. The DRIVE database consists of a set of 40 RGB color fundus photographs obtained from a diabetic retinopathy screening program^17^. The images (565 × 584 pixels) were acquired using a Canon CR5 non-mydriatic 3-CCD camera with a FOV of 45^o^. DRIVE database is divided into two sets, the training set and the test set, each containing twenty images. The test set was manually segmented by two observers and the first observer is accepted as ground truth. The STARE database contains a set of twenty images, ten of which show signs of pathology^15^. The images (700 × 605 pixels, 8-bit per color channel) were captured using a TopCon TRV-50 fundus camera with a FOV of 35^o^. Two experts manually segmented all images and the first observer is regarded as ground truth.

The vessel width measurement method was evaluated on the REVIEW database, which is widely used for the evaluation of vessel width measurement methods^28^. The REVIEW database contains four image sets with vessel measurement from three observers. The VDIS dataset contains severe disease cases and the KPIS dataset includes only parts of retinal images. These two datasets were thus excluded in this study. The other two datasets (i.e., HRIS and CLRIS) were included for validation. The HRIS dataset represents different stage of DR and consists of 2368 manual vessel profiles from 90 vessel segments. The CLRIS dataset includes a strong central light reflex phenomenon and contains 285 vessel profiles from 21 vessel segments. Each profile in the REVIEW database consists of fifteen numbers: series number, image number, segment number, and the coordinates of the left and right boundaries (*x*_*1*_, *y*_*1*_, *x*_*2*_, *y*_*2*_) from three observers (*O*_*1*_, *Ο*_*2*_, and *O*_*3*_).

### Clinical data

The clinical images were collected at Xi’an No.1 Hospital. Ten normal eyes from ten participants were included in this study. We captured both high quality fundus images using a DF-camera (Topcon TRC-NW8 fundus camera, FOV of 45^o^) and low quality fundus images using a HF-camera (iExaminer plus ‘Panoptic’, FOV of 25^o^, Welch-Allyn Inc., Skaneateles Fall, NY, USA)^10^). The low quality images showed a smaller FOV, lower resolution, and poorer contrast due to the hardware limitations, greatly increasing the difficulties of image analysis. Informed consent for research use of data was sought and obtained from each study participant before participation. The study was approved by the Ethics Committee of Xi’an Jiaotong University. The methods were carried out in accordance with the approved guidelines.

### Visual saliency based vessel segmentation

The vessel analysis system contains a vessel segmentation algorithm and a vessel width measurement algorithm. Vessel segmentation is the fundamental task for further analyses of retinal vasculature, such as vessel width, vessel tortuosity, branching angle, and arteriovenous ratio. In this study, a fast and accurate vessel segmentation algorithm based on visual saliency is introduced. One advantage of saliency based method is that only global operators and linear local neighborhood operators are used, meaning it is simple in algorithmic design and fast in computational performance. In this method, multi-scale salient features are generated, including spectral residual, orientation, morphological features, and self-information. When all four salient features are extracted, a gray scale vessel image is created by a linear combination of all normalized saliency features. A binary vessel image is created using Triangle thresholding. More details are given in Supplementary Materials.

### Graph-theoretic vessel width measurement

We previously reported a vessel width measurement method based on three-dimensional graph search, which converts the simultaneous two-boundary detection problem into a two-slice three-dimensional minimum closed set problem^29^. This method achieves high accuracy but suffers from high computation complexity because of the three-dimensional graph design. Here, we improved this method by breaking the three-dimensional graph down to two separate two-dimensional graphs. To do this, the inter-slice smoothness constraint, *i.e.*, the connection between the two slices, is removed and the intra-slice smoothness constraint is strengthened. In this way, the computational performance can be greatly improved with limited sacrifice in accuracy. After the two-dimensional graphs are constructed, they are solved as separate minimum closed set problems. Once both vessel boundaries are solved, the vessel width is determined as the distance between the left boundary and the right boundary along the normal direction of the blood vessel. More details are given in Supplementary Materials.

### Smartphone-based GUI system

We developed an Android app as a graphical user interface (GUI). Specifically, this app is designed for multiple tasks. (a) account management;(b) loading fundus images;(c) fully automatic image analyses;(d) result display, storage, and up-linking;(e) user instruction. The details are described below:

- *Account management*: this system allows multiple users on a single smartphone, each of which is protected by a user name and a password.
- *Loading fund images*: the system allows loading fundus images from the smartphone gallery or capturing new fundus images if the smartphone is equipped with a portable fundus camera.
- *Automatic image analyses:* the system allows automatic analyses of a fundus image, including vessel segmentation and width measurement as described above.
- *Result display, storage, and up-linking:* both the visualized result and quantitative result can be displayed, stored, or emailed to a professional personnel.
- *User instruction:* the system provides detailed instruction on how to perform a new test and how to understand the results.

### Statistical analysis

Statistical analysis was performed using the Statistical Package for the Social Sciences (SPSS ver. 19.0, Chicago, IL, USA). For vessel segmentation, SN, SP, Acc, and ROC curve were calculated and compared with other reported methods. SN is defined as TP/(TP + FN), reflecting the ability of an algorithm to find vessel pixels, where TP means true positives and FN means false negative. SP is defined as TN/(TN + FP), reflecting the ability of an algorithm to find non-vessel pixels, where TN means true negative and FP means false positive. Acc is defined as the ratio of the total number of correctly classified pixels (TP + TN) to the total number of pixels in the image. The ROC curve shows the tradeoff between sensitivity and specificity as its discrimination threshold varies. The closer an ROC curve approaches the top left corner, the better the performance of the method. The AUC, ranging from 0 to 1, reflects the overall performance of the method, in which a perfect test has an AUC of 1.

In the vessel width measurement, for each measurement from each observer, the vessel centerline is defined as 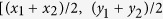 and vessel width as 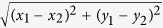. A reference standard (RS) is created by averaging the manual measurements from the three observers. Comparison between different methods was performed by five parameters: success rate, mean and standard deviation of average vessel width, and mean and standard deviation of individual differences. The success rate is defined as the ratio between matched points and total RS centerline points, in which a matched is defined as success if at least one detected centerline pixel is found within certain distance to an RS centerline point. The mean and standard deviation of the average vessel width reflect the overall similarity between different methods while the mean and standard deviation of the individual differences reflect the overall differences between individual measurements.

In the validation on low quality clinical images, the paired student’s *t*-test was used to evaluate the difference between the vessel widths from high quality image and low quality image. A *p* value of < 0.05 was considered statistically significant. The scatter plot and Pearson’s correlation were also reported.

### Platform

The proposed methods were implemented in Android 4.2.2. Java library OpenCV was included for basic image processing and library JGraphT was used for basic graph construction and solution. All tests were performed on a Samsung Galaxy S5 smartphone (Qualcomm snapdragon 801, 2.5GHz, RAM 2 GB).

## Additional Information

**How to cite this article**: Xu, X. *et al*. Smartphone-Based Accurate Analysis of Retinal Vasculature towards Point-of-Care Diagnostics. *Sci. Rep.* **6**, 34603; doi: 10.1038/srep34603 (2016).

## Supplementary Material

Supplementary Information

## Figures and Tables

**Table 1:** Comparative performance of different segmentation methods on the DRIVE and STARE databases.

Data	DRIVE Test					STARE					Platform
Methods	Acc	SP	SN	AUC	Time	Acc	SP	SN	AUC	Time	
Human	0.947	0.972	0.776	—	—	0.935	0.938	0.895	—	—	—
Niemeijer *et al*.^17^	0.942	0.969	0.689	0.930	—	—	—	—	—	—	—
Hoover *et al*.^15^	—	—	—	—	—	0.9275	0.81	0.65	—	5min	SunSPARCstation 20
Jiang *et al*.^18^	0.891	0.90	0.830	0.932	8–36s	0.901	0.90	0.857	0.929	8–36s	600 MHz PC
Staal *et al*.^19^	0.944	0.977	0.719	0.952	15min	0.952	0.981	0.697	0.961	15min	1.0 GHz, 1GB RAM
Mendonca *et al*.^20^	0.945	0.976	0.734	—	2.5min	0.944	0.973	0.699	—	3min	
Soares *et al*.^30^	0.946	0.978	0.733	0.961	~3min	0.948	0.975	0.72	0.967	~3min	2.17GHz, 1GB RAM
Ricci *et al*.^21^	0.959	0.972	0.775	0.963	—	0.965	0.939	0.903	0.968	—	—
Al—Diri *et al*.^22^	—	0.955	0.728	—	11min	—	0.968	0.752	—	—	1.2 GHz
Marin *et al*.^13^	0.945	0.98	0.706	0.958	~90s	0.952	0.982	0.694	0.977	~90s	2.13GHz, 2GB RAM
Fraz *et al*.^31^	0.948	0.981	0.74	0.974	~100s	0.953	0.976	0.755	0.976	~100s	2.27GHz, 4GB RAM
Lam *et al*.^23^	0.947	—	—	0.961	13min	0.957	—	—	0.974	13min	1.83GHz, 2GB RAM
Budai *et al*.^32^	0.957	0.987	0.644	—	~5s	0.938	0.982	0.58	—	~6s	2.3 GHz, 4GB RAM
Perez *et al*.^33^	0.925	0.967	0.644	—	~2min	0.926	0.944	0.769	—	~2min	Parallel Cluster
Miri *et al*.^34^	0.943	0.976	0.715	—	~50s	—	—	—	—	—	3 GHz, 1 GB RAM
Roychowdhury *et al*.^24^	0.949	0.978	0.739	0.967	2.45s	0.956	0.984	0.732	0.967	3.95s	2.6 GHz, 2GB RAM
**Proposed**	**0.933**	**0.955**	**0.786**	**0.959**	**~118s**	**0.920**	**0.931**	**0.825**	**0.959**	**~130s**	**Android Samsung Galaxy S5**

**Table 2:** Comparison of vessel width measurement methods on HRIS dataset.

Method Name	Success Rate %	Measurement		Difference	
		μ	σ	μ	σ
Observer 1	100	4.12	1.25	−0.23	0.29
Observer 2	100	4.35	1.35	0.002	0.26
Observer 3	100	4.58	1.26	0.23	0.29
Gregson’s Algorithm^25^	100	7.64	—	3.29	2.84
Half-height full-width (HHFW)^35^	88.3	4.97	—	0.62	0.93
1D Gaussian Model-fitting^36^	99.6	3.81	—	−0.54	4.14
2D Gaussian Model-fitting^37^	98.9	4.18	—	−0.17	6.02
Extraction of Segment Profiles^22^	99.7	4.63	—	0.28	0.42
3D Graph-Based Method^29^	100	4.56	1.30	0.21	0.57
2D Graph-Based Method	94.0	4.16	1.20	−0.18	0.70

**Table 3:** Comparison of vessel width measurement methods on CLRIS dataset.

Method Name	Success Rate %	Measurement		Difference	
		μ	σ	μ	σ
Observer 1	100	13.19	4.01	−0.61	0.57
Observer 2	100	13.69	4.22	−0.11	0.70
Observer 3	100	14.52	4.26	0.72	0.57
Gregson’s Algorithm^25^	100	12.8	—	−1.0	2.84
Half-height full-width (HHFW)^35^	0	—	—	—	—
1D Gaussian Model-fitting^36^	98.6	6.3	—	−7.5	4.14
2D Gaussian Model-fitting^37^	26.7	7.0	—	−6.8	6.02
Extraction of Segment Profiles^22^	93.0	15.7	—	−1.9	1.50
3D Graph-Based Method^29^	94.1	14.05	4.47	0.08	1.78
2D Graph-Based Method	93.4	13.84	4.82	0.04	1.89
