# Supplementary material for: Smartphone-Based Accurate Analysis of Retinal Vasculature towards Point-of-Care Diagnostics
Source: Sci Rep. 2016 Oct 4;6:34603. doi: 10.1038/srep34603 (PMC5048171; doi:10.1038/srep34603)
Supplement: Supplementary Information [file srep34603-s1.pdf]

Supplementary Information

Smartphone-Based Accurate Analysis of Retinal Vasculature towards Point-of-Care Diagnostics

Xiayu Xu[a,b], Wenxiang Ding[a,b], Xuemin Wang[a,b], Ruofan Cao[a,b], Maiye Zhang[c], Peiling Lv[a,b], Feng Xu[a,b*]

[a] *The Key Laboratory of Biomedical Information Engineering, Ministry of Education, School of Life Science and Technology, Xi'an Jiaotong University, Xi'an 710049, P.R. China*

[b] *Bioinspired Engineering and Biomechanics Center (BEBC), Xi'an Jiaotong University, Xi'an 710049, P.R. China*

[c] *Department of Endocrinology and Metabolism, Fourth Military Medical University, 169 West Changle Road, Xi'an, Shaanxi 710032, P. R. China*

[*] *Corresponding author: fengxu@mail.xjtu.edu.cn*

1. Visual salient feature extraction

1.1 Preprocessing

First of all, the central light reflex (CLR), which is caused by specular reflection and appears as a bright streak running down the center of blood vessels, is removed. Similarly, fundus images also commonly suffer from background intensity variation caused by uneven illumination on the spherical surface of retina, meaning retinal blood vasculature may show different intensity across the whole image and will results in inhomogeneous characteristics in the salient images. Hence, before extracting the saliency features, a background adjustment algorithm is performed.

The central light reflex is removed by applying a gray scale opening operation with a kernel size of three pixel (**Eq. 1**). The red, green, and blue channels are processed in a similar way respectively.

$$A \circ B = (A \ominus B) \oplus B \tag{1}$$

where $\ominus$ and $\oplus$ are the erosion and dilation operations in image morphological processing. To adjust the uneven background, a Gaussian filter with a large kernel is convolved with the original image to remove high frequency information on the image, leaving only the slowly changing background information, which is then subtracted from the original image. The equations are given below:

$$R(x, y) = I(x, y) - \varphi(x, y) * I(x, y) \tag{2}$$

$$\varphi(x, y) = \frac{1}{2\pi\sigma} \exp\left(-\frac{x^2 + y^2}{2\sigma^2}\right) \tag{3}$$

where φ is a Gaussian filter.

1.2 Visual saliency features extraction.

1.2.1 Multi-scale spectral residual feature

The spectral residual is calculated to remove the low frequency information, such as background, and keep only the high frequency information, such as blood vessels [1]. In order to preserve blood vessels with different calibers, multiscale strategy is used. First of all, the number of scale levels is first calculated as:

$$n = \frac{\log_2 l}{2} - 1 \tag{4}$$

where l is the shorter border of the image. The zero level image I_0 is defined as the Gaussian blurred original intensity image and the next level pyramid is generated by a down-sample of the higher level image followed by a Gaussian blur.

$$I_0(x,y) = I(x,y) * \varphi(x,y) \tag{5}$$

$$\begin{cases} I'_{\delta+1} = I_\delta(2x,2y) \\ I_{\delta+1}(x,y) = I'_{\delta+1}(x,y) * \varphi(x,y) \end{cases}, \delta \in [0,...,n-1] \tag{6}$$

$$\varphi(x,y) = \frac{1}{2\pi\sigma}\exp(-\frac{x^2+y^2}{2\sigma^2}) \tag{7}$$

where $*$ denotes the convolution operation.

For each level, the Gaussian blurred intensity image is used to calculate the spectral residual as follows:

$$F_\delta(u,v) = \int_{-\infty}^{\infty}\int_{-\infty}^{\infty} I_\delta(x,y)e^{-2\pi(xu+yv)}dxdy = |A_\delta(u,v)|e^{i\varphi_\delta(u,v)} \tag{7}$$

where $A_\delta(u,v)$ is the amplitude of the Fourier transform and $\varphi_\delta(u,v)$ is the phase. The spectral residual $R_\delta(u,v)$ is calculated as the difference between amplitude in logarithm scale and the blurred amplitude in logarithm scale (**Eq. 8**). The feature image is derived by applying an inverse Fourier transform followed by a Gaussian filter.

$$R_\delta(u,v) = \log\left(A_\delta(u,v)\right) - \log\left(A_\delta(u,v)\right) * h \tag{8}$$

$$F'_\delta(u,v) = \exp\left(R_\delta(u,v)\right)e^{i\varphi_\delta(u,v)} \tag{9}$$

$$f_\delta(x,y) = \int_{-\infty}^{\infty}\int_{-\infty}^{\infty} I_\delta(x,y)e^{-2\pi(xu+yv)}dxdy \tag{10}$$

$$\hat{I}_\delta(x,y) = \varphi(x,y) * f_\delta(x,y) \tag{11}$$

where h is an average filter. After all feature maps at different scale are obtained, a weighted across-scale fusion method is applied to combine the feature maps into a single spectral residual feature image. Example original fundus images from the

DRIVE and STARE databases are given in **Supplemental Fig. Ia** and **IIa** and the spectral residual feature images are given in **Supplemental Fig. Ib** and **IIb**.

$$\breve{I} = \sum_{\sigma=0}^{n} W[I_{\delta}(x,y)] \tag{12}$$

where W is a weight that proportionate to the quadratic inverse mean intensity of each feature map. All images are up-sampled to level 0 before the image fusion.

1.2.2 Morphological feature

Retinal blood vessels can be seen as dark object on a relatively bright background. To extract the morphological features, a grayscale bottom-hat operation is applied (**Supplemental Fig. Ic** and **IIc**):

$$B_{hat}(f) = (f \oplus b) \ominus b - f \tag{13}$$

where $f(x,y)$ denotes a gray-scale image and $b(x,y)$ is a structuring element.

1.2.3 Orientation feature

Retinal blood vessels can be seen as multi-oriented elongate structures and Gabor wavelets have good performance in direction selection and fine tuning to specific frequencies [2]. A 2D Gabor filter can be seen as a complex exponential modulated Gaussian (**Eq. 14**).

$$h(\hat{x},\hat{y},\theta) = \frac{1}{2\pi\sigma_x\sigma_y}\exp\left[-\frac{1}{2}\left(\frac{\hat{x}^2}{\sigma_x^2}+\frac{\hat{y}^2}{\sigma_y^2}\right)\right]\exp\left[2\pi i W_{\hat{x}}\right] \tag{14}$$

$$\begin{cases} \hat{x} = x\cos(\theta) + y\sin(\theta) \\ \hat{y} = -x\sin(\theta) + y\cos(\theta) \end{cases} \tag{15}$$

The sigma values are 5 and 9. The kernel is rotated every twenty degrees and results in a set of nine kernels in total. The real part of a Gabor filter is even and sensitive to oriented edges. The imaginary part is odd and sensitive to oriented lines. After a study on both filters in this study, we chose the imaginary Gabor kernel only. After convolution with the Gabor kernel, the absolute values of the negative pixels are

selected. To attain the final orientation feature, the maximum response of each pixel is regarded as the final response (**Supplemental Fig. Id** and **IId**).

1.2.4 Self-information feature

The feature based on self-information theory is also explored in this study. By definition, the amount of self-information contained in a probabilistic event depends only on the probability of that event, *i.e.*, the smaller its probability, the larger the self-information associated with receiving the information that the event indeed occurred. Following this definition, retinal blood vessels, which is a minority event comparing with background event, contained more self-information. The panchromatic and multispectral channels are used to calculate the self-information feature. The panchromatic channel is calculated as the average of red, green, and blue channels and the self-information is calculated as:

$$I(\omega_n) = -\log\left(P(\omega_n)\right) \tag{16}$$

where ω_n is an independent event (the intensity of a given pixel in an image in this case) and $P(\omega_n)$ is the probability of ω_n (the corresponding value of ω_n in the normalized histogram in this case). The histogram and normalized histogram is calculated as below:

$$h(r_k) = n_k, k = 0,1,2,...,L \tag{17}$$

$$p(r_k) = \frac{n_k}{M \times N}, k = 0,1,2,...,L \tag{18}$$

where L is the maximum gray value in the image, M and N are the height and width of the image. Then the self-information contained in each intensity level is calculated as in **Eq. 16** and the intensity of each pixel is replaced with the self-information:

$$E_l(i,j) = I\left(A_l(i,j)\right) \tag{19}$$

The final self-information feature image is calculated as the weighted average of panchromatic and red, green, and blue channels (**Supplemental Fig. Ie** and **IIe**).

$$\breve{E} = \sum_{l=1}^{4} w_l E_l \quad (20)$$

$$w_l = -\log\left(\frac{h_1}{h_1 + h_2 + h_3 + h_4}\right) \quad (21)$$

where h_1, h_2, h_3, and h_4 are total pixel value of panchromatic and red, green, and blue channels respectively.

1.2.5 Feature fusion

The intensity, morphological, orientation, and self-information are combined to build the final saliency image (**Eq.22**).

$$S = \sum_{n=1}^{4} w_n E_n \quad (22)$$

where S is the saliency image, w is the weight of each feature image calculated as the reciprocal of mean intensity of each feature map. A binary vessel image is created using Triangle thresholding (**Supplemental Fig. If** and **IIf**).

2. Graph-theoretic vessel width measurement

The two-dimensional graph method is an improvement of the previously reported three-dimensional graph method [3]. The three-dimensional graph method achieves high accuracy but suffers from high computational complexity because of the three-dimensional graph design. In this work, in order to improve the computational performance, the three-dimensional graph is broken down to two separate two-dimensional graphs. **The vessels within the optic disc are currently included and will be excluded when specific parameters, such as the arteriovenous ratio, are calculated in the future.**

To build the two-dimensional graph, the vessel centerline image is generated through sequential thinning of the vessel segmentation created above (**Supplementary Fig. 2a**). First of all, the bifurcation points and crossing points are detected and removed to cut the vessel trees into separate vessel segments for further graph construction. For

each vessel centerline pixel p_i, $i \in (n, l\text{-}n)$ on a vessel segment, the vessel growing direction is found as the direction pointing from n pixels left from p_i to n pixels right from p_i, where l is the length of the vessel segment. For end points that do not have enough neighboring pixels on one side, the direction of the nearest centerline pixel that has a definition of direction is assigned. Vessel segment with $l<2n+1$ is excluded for further graph construction. After the vessel growing direction is found for each centerline pixel, the counter-clockwise 90° is regarded as the normal direction at this point.

For each vessel segment, the positive normal direction side is constructed as one graph (G_1) and the negative normal direction side is constructed as another graph (G_2) (**Supplementary Fig. 2b**). Starting from each vessel centerline p_i and along the positive normal direction, graph nodes $N(i, j)$ are generated with a step of 0.5 pixels. Inter-column arcs are assigned from $N(i, j)$ to $N(i\text{-}1, \max(0, j\text{-}\Delta x)) \in Col(i\text{-}1)$ and from $N(i, j) \in Col(i)$ to $N(i\text{+}1, \max(0, j\text{-}\Delta x)) \in Col(i\text{+}1)$, with parameter Δx being the maximum difference allowed between two adjacent normal profiles within one boundary. **The maximum difference allowed between two adjacent profile Δx is fixed to one graph node to maintain the smoothness of the vessel boundary.** A solution is feasible if it satisfies the smoothness constraint defined by Δx. **The cost image is generated from the orientation sensitive first-order derivative of Gaussian of the green channel.**

$$f(x;\sigma) = -\frac{x}{\sigma^3\sqrt{2\pi}}\exp\left(-\frac{x^2}{2\sigma^2}\right) \qquad \textbf{(23)}$$

where σ is scale-sensitive and is calculated by rounding up the product of 0.002 and the shorter boundary of the image. After the node-weighted graph is constructed, it is solved as a minimum closed set problem (**Supplementary Fig. 2c**) [4]. At last, the vessel width is defined as the distance between the outmost node of the closed set in G_1 and the outmost node of the closed set in G_2 along the graph column (**Supplementary Fig. 2d**).

Supplementary Figure 1. Visualized results of vessel segmentation. First two rows are the original fundus image, intensity feature, morphological feature, orientation feature, spectral feature, and final vessel segmentation of a sample image from DRIVE database. Last two rows are the original fundus image, intensity feature, morphological feature, orientation feature, spectral feature, and final vessel segmentation of a sample image from STARE database.

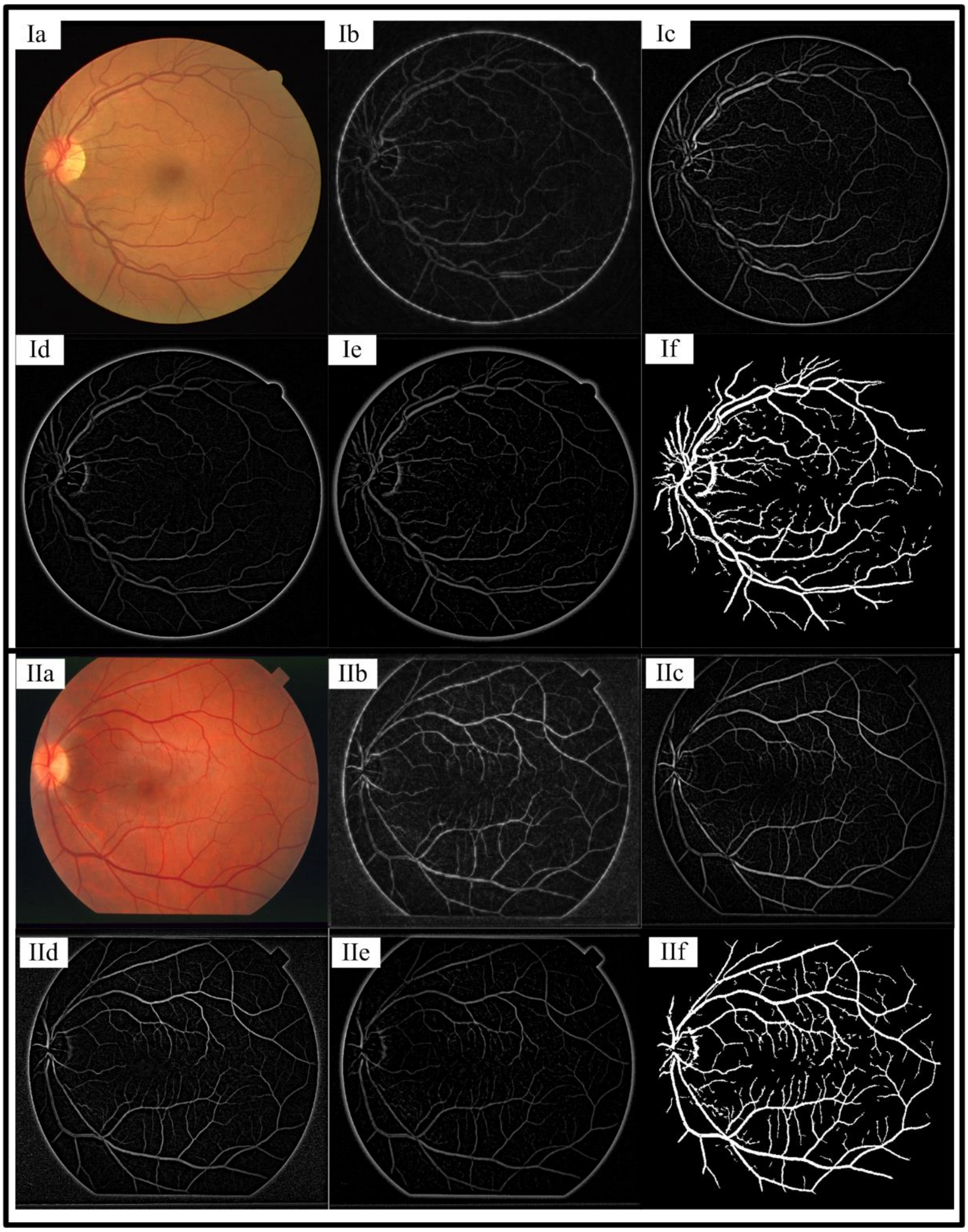

Supplementary Figure 2. An illustration of graph construction. (a) First of all, the blood vessels are detected from the original fundus image, after which the vessel centerline is extracted for each vessel segment. (b) Two node-weighted graphs, G_1 and G_2, are constructed. The graph columns are along the normal direction of the vessel growing direction. The dash lines indicate the target vessel boundaries. (c) The node-weighted graphs are solved as minimum closed set problem. The green nodes indicated the closed sets. (d) The vessel width is determined as the distance between the green nodes along each column.

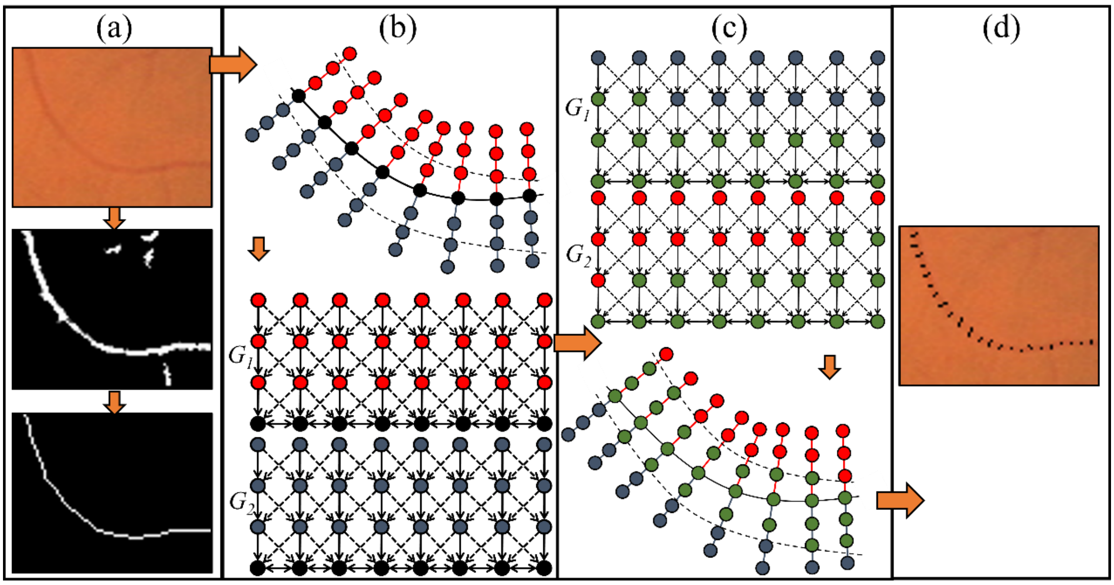

1. Zhang, L., et al., *Multi-scale hybrid saliency analysis for region of interest detection in very high resolution remote sensing images.* Image and Vision Computing, 2014.
2. Soares, J.V., et al., *Retinal vessel segmentation using the 2-D Gabor wavelet and supervised classification.* Medical Imaging, IEEE Transactions on, 2006. **25**(9): p. 1214-1222.
3. Xu, X., et al., *Vessel boundary delineation on fundus images using graph-based approach.* Medical Imaging, IEEE Transactions on, 2011. **30**(6): p. 1184-1191.
4. Li, K., et al., *Optimal surface segmentation in volumetric images-a graph-theoretic approach.* Pattern Analysis and Machine Intelligence, IEEE Transactions on, 2006. **28**(1): p. 119-134.
